# Should we search Chinese biomedical databases when performing systematic reviews?

**DOI:** 10.1186/s13643-015-0017-3

**Published:** 2015-03-06

**Authors:** Jérémie F Cohen, Daniël A Korevaar, Junfeng Wang, René Spijker, Patrick M Bossuyt

**Affiliations:** Department of Clinical Epidemiology, Biostatistics and Bioinformatics, Academic Medical Center - University of Amsterdam, Room J1B-210, PO Box 22700, 1100 DE Amsterdam, The Netherlands; Inserm U1153, Obstetrical, Perinatal and Pediatric Epidemiology Research Team, Center for Epidemiology and Biostatistics Sorbonne Paris Cité (CRESS), Paris Descartes University, 53 Avenue de l’Observatoire, 75014 Paris, France; Department of Pediatrics, Necker-Enfants Malades Hospital, Assistance Publique-Hôpitaux de Paris, Paris Descartes University, 149 Rue de Sèvres, 75015 Paris, France; Julius Center for Health Sciences and Primary Care, University Medical Center Utrecht, Dutch Cochrane Centre, Heidelberglaan 100, 3584 CX Utrecht, The Netherlands

**Keywords:** Systematic reviews, Literature searching, Information retrieval, Bias, Language of publication, China, Databases

## Abstract

**Background:**

Chinese biomedical databases contain a large number of publications available to systematic reviewers, but it is unclear whether they are used for synthesizing the available evidence.

**Methods:**

We report a case of two systematic reviews on the accuracy of anti-cyclic citrullinated peptide for diagnosing rheumatoid arthritis. In one of these, the authors did not search Chinese databases; in the other, they did. We additionally assessed the extent to which Cochrane reviewers have searched Chinese databases in a systematic overview of the Cochrane Library (inception to 2014).

**Results:**

The two diagnostic reviews included a total of 269 unique studies, but only 4 studies were included in both reviews. The first review included five studies published in the Chinese language (out of 151) while the second included 114 (out of 118). The summary accuracy estimates from the two reviews were comparable. Only 243 of the published 8,680 Cochrane reviews (less than 3%) searched one or more of the five major Chinese databases. These Chinese databases index about 2,500 journals, of which less than 6% are also indexed in MEDLINE. All 243 Cochrane reviews evaluated an intervention, 179 (74%) had at least one author with a Chinese affiliation; 118 (49%) addressed a topic in complementary or alternative medicine.

**Discussion and conclusions:**

Although searching Chinese databases may lead to the identification of a large amount of additional clinical evidence, Cochrane reviewers have rarely included them in their search strategy. We encourage future initiatives to evaluate more systematically the relevance of searching Chinese databases, as well as collaborative efforts to allow better incorporation of Chinese resources in systematic reviews.

## Background

Systematic reviews are a cornerstone of evidence-based medicine. Developing a comprehensive literature search is a key element in retrieving all relevant evidence available to answer a specific clinical question. Since it was shown that trials with significant results are more likely to be published in English-language journals [1], standards from the Cochrane Collaboration recommend that ‘whenever possible review authors should attempt to identify and assess for eligibility all possibly relevant reports of trials irrespective of language of publication’ [2]. Including studies published in languages other than English not only prevents from language bias but also increases precision of meta-analysis estimates and statistical power to explore sources of heterogeneity.

Several empirical studies have evaluated the influence of English language restrictions in systematic reviews and meta-analyses of treatment effects [3]. A systematic review of those studies found no evidence of bias when restricting to English language literature in reviews of conventional medical interventions [3]. A similar evaluation in the field of complementary and alternative medicine found that English-language studies tend to report smaller effects than those published in other languages [4].

The Chinese biomedical literature has been continuously growing over the past decades. China’s share in the world’s total published scientific papers increased from less than 1% in 1980 to about 12% in 2011, currently ranking second behind the US [5]. There is limited evidence that searching Chinese databases when performing systematic reviews leads to the identification of additional relevant studies and influences the outcome of meta-analysis. Available studies are limited to the field of traditional Chinese medicine, and the results of such investigations have been conflicting [6-8].

We here report on a case of two recently published independent systematic reviews on the same topic: one for which the authors did not search in Chinese databases and the other for which the authors did. We describe five major Chinese electronic biomedical literature databases, with regard to content, search features, and accessibility. We also evaluate to what extent Cochrane reviewers have included these Chinese databases in their search strategy.

## Main text

### Case report

In an ongoing meta-epidemiological project about methods used in systematic reviews of diagnostic accuracy studies [9,10], we identified a systematic review on the accuracy of anti-cyclic citrullinated peptide (anti-CCP) antibodies for diagnosing rheumatoid arthritis [11]. We were also aware of another systematic review on the same topic in which former members of our research group participated [12]. In both reviews, the 1987 revised American College of Rheumatology criteria served as the clinical reference standard [11,12]. A detailed description of the reviews can be found in Table 1. Both reviews covered the same topic and showed overlap in their timeframe of interest. We additionally identified two earlier systematic reviews on the same topic, but they could not be compared with the others because of different timeframes for the literature search [13,14].

**Table 1:** **Comparison of Whiting** ***et al*** **.’s and Gao** ***et al*** **.’s systematic reviews**

**Review characteristic**	**Whiting** ***et al.*** **2010** [12]	**Gao** ***et al.*** **2012** [11]
Primary aim	To compare the accuracy of anti-CCP antibodies and rheumatoid factor in diagnosing rheumatoid arthritis in patients with early symptoms of the disease	To determine the accuracy of anti-CCP antibody in diagnosis of patients with rheumatoid arthritis in a Chinese population
Databases searched	MEDLINE, Embase, Science Citation Index, BIOSIS, ISI Web of Science, System for Information on Grey Literature in Europe, Zetoc, National Technical and Information System, Dissertation Express	MEDLINE, CNKI
Timeframe for searches	Inception to September 2009	January 2000 to June 2010
Language restrictions	None	English and Chinese
Number of studies included		
Total	151^a^	118
English language	130	4
Chinese Language	5	114
Other non-English languages	20	0
Summary estimate of sensitivity (95% CI)	68% (65 to 71)^b^	65% (65 to 66)
Summary estimate of specificity (95% CI)	95% (94 to 96)^b^	95% (95 to 96)

^a^151 studies corresponding to 155 publications were included; ^b^Estimates from case-control studies (*n* = 97). Anti-CCP, anti-cyclic citrullinated peptide; CI, confidence interval; CNKI, China National Knowledge Infrastructure.

In contrast with the review of Whiting *et al*., the review from Gao *et al*. specifically aimed to evaluate the accuracy of anti-CCP antibodies in the Chinese population. The rationale for restricting the review to the Chinese population was a presumed variability in anti-CCP accuracy, related to the genetic or ethnic background. Gao *et al*. only searched for studies reported in English or Chinese, whereas the review from Whiting *et al*. had no geographical or language restrictions.

**Figure 1:**
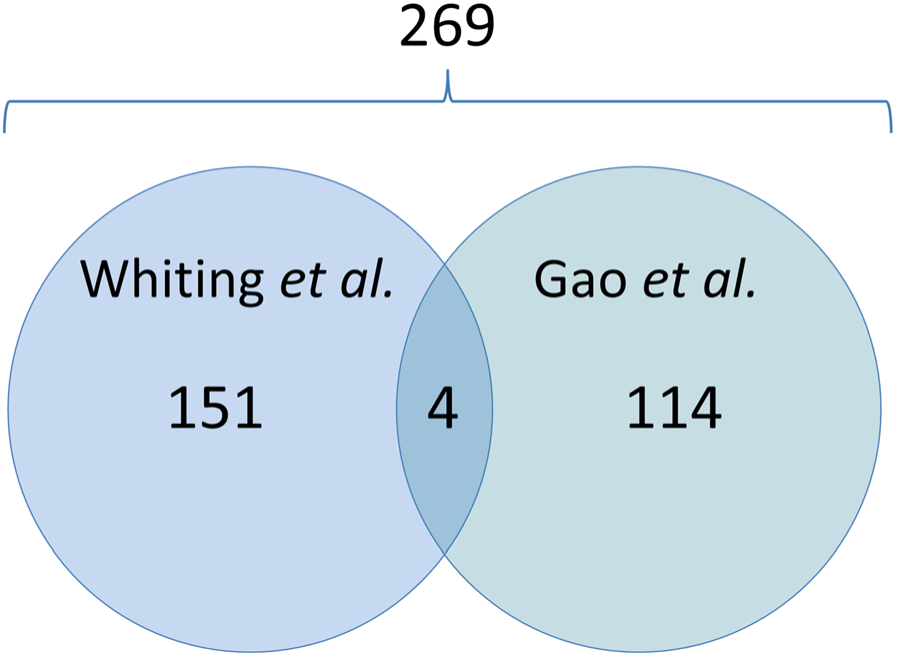
**Number of studies included by each review and corresponding overlap.**

The most striking finding when comparing the two reviews is the large number of studies published in the Chinese language identified by Gao *et al*. but not by Whiting *et al*. Gao and colleagues searched two biomedical databases (MEDLINE and China National Knowledge Infrastructure (CNKI), 2000 to 2010) and included 118 studies, of which 114 had been published in the Chinese language. Although Whiting and her colleagues thoroughly searched 10 databases (inception to 2009) without language restrictions and were able to include 151 studies (155 publications), only 5 of these were reported in the Chinese language. In total, the two reviews identified 269 unique studies. Surprisingly, only four primary studies can be found in both reviews (Figure 1). This discrepancy can be explained by the fact that Gao *et al*. also searched CNKI, one of the largest Chinese biomedical databases, while Whiting *et al*. did not.

The summary estimates of test accuracy generated by the two meta-analyses were comparable. Gao *et al*. reported a summary estimate of sensitivity of 65% (95% confidence interval (CI) 65% to 66%) at a specificity of 95% (95% to 96%). Most studies in this review had used multiple sets of patient inclusion criteria. Whiting *et al*. analyzed a subset of case-control studies in their review (n = 97) and reported summary estimates of 68% (65% to 71%) for sensitivity and 95% (94% to 96%) for specificity.

A limitation when trying to compare the two anti-CCP reviews lies in the lack of details about the index tests under investigation in Gao’s review. Whiting *et al.* provided a complete description of the different types of anti-CCP antibodies evaluated across primary studies (antifilaggrin antibodies; antikeratin antibodies; antiperinuclear factor; first, second, or third generation anti-CCP; mutated citrullinated vimentin) whereas Gao *et al*. only characterized assays by manufacturer (Eurimmun vs. non-Eurimmun tests). Yet the fact that the summary accuracy estimates are almost identical in both reviews suggests that the same kind of index tests were reviewed.

In this case, a very large body of evidence (114 studies) was missed through not searching in a Chinese database, but this additional evidence seemed to be of no influence on summary accuracy estimates. Here, the results were similar and it may therefore be unnecessary to search Chinese databases, but no claim is made that this would apply to all cases. It is largely unknown whether Chinese databases contain relevant clinical data for systematic reviews and whether including this data would affect the outcomes and conclusions. Further research is needed to resolve this question, especially in fields other than complementary and alternative medicine. Wu *et al*. previously reported on a case of two independent reviews evaluating the effectiveness of acupuncture for chronic asthma [8]. While the first review only searched English language databases, the other also searched Chinese databases. This strategy almost doubled the number of included trials (12 vs. 22) and led to an almost ninefold increase in the number of included patients (350 vs. 3,058). The review that searched in Chinese databases found a significant effect of acupuncture on spirometry parameters whereas the other review found no evidence favoring acupuncture. It is highly likely that the amount of data in Chinese databases and their effect on the summary estimates differ by topic of interest and across study types (for example, interventions, diagnostic test accuracy, and prognostic and omics marker evaluations).

## Chinese biomedical databases

Xia *et al*. have extensively described five major biomedical databases originating from China: CNKI, Chinese Biomedical Literature (CBM), Chinese Medical Current Content (CMCC), VIP, and WANFANG (China Online Journals) [15]. They reported that less than 6% of the 2,500 journals indexed in these databases were also indexed in MEDLINE. All databases had advanced search features that allowed combining keywords with Boolean and proximity operators. Four databases had even more advanced search features that allowed command-line-style queries with keywords, fields, Boolean, and proximity operators. All databases accepted English-written queries but all were also language sensitive. In terms of accessibility, all databases but CBM gave free access to study abstracts and offered the possibility to purchase full-text articles without full subscription to the database. CBM search features were accessible to subscribers only. In another recent evaluation of the relevance of searching Chinese biomedical databases (CBM, CNKI, VIP, and WANFANG) when conducting systematic reviews, Ai *et al*. found that CBM and CNKI were the two databases that covered the most journals (1,784 and 1,126, respectively) [16]. The authors recommended CBM for reviewers who aim to search for studies in Chinese and CNKI for non-Chinese-speaking researchers.

## Searching Chinese databases: current practice among Cochrane reviewers

The Cochrane Collaboration recommends searching without language restrictions to avoid language bias. It also suggests searching regional databases when relevant, citing CBM as an example of a Chinese database [2]. We aimed to evaluate whether authors of published Cochrane reviews had searched Chinese databases.

Study selection was performed by searching the full text of Cochrane reviews for any reference to at least one of the five major Chinese databases. On October 8, 2014, we searched the Cochrane Library from inception using full text screening for the words ‘China National Knowledge Infrastructure’, ‘China Academic Journals’ (former name of CNKI), ‘cnki’, ‘Chinese Biomedical Literature Database’, ‘cbm’, ‘Chinese Medical Current Content’, ‘cmcc’, ‘VIP’, ‘Wanfang’, or ‘Wan fang’. Protocols were excluded. Data extraction was performed by one author (JFC or DAK) using a standardized form. We extracted the name of the first author and publication year, whether the reviewers had applied any language restrictions, which of the five Chinese databases were searched, and whether any review author had a Chinese affiliation. We also extracted whether the review addressed a complementary or alternative medicine topic (as defined by Kemper *et al*.) [17].

Among 8,680 published reviews indexed in the Cochrane Library, only 243 (3%) had searched at least one of the five major Chinese databases. These were retained for further analysis. All included Cochrane reviews evaluated an intervention, 179 (74%) had at least one author with a Chinese affiliation and 118 (49%) addressed a topic in complementary or alternative medicine. About half of reviewers had searched multiple Chinese databases (135, 56%). CNKI and CBM were most frequently searched (in 185 [76%] and 162 [67%] cases, respectively) while CMCC was searched in only 21 cases (9%). The great majority of the reviews, 213 (88%), explicitly declared not to have applied any language restrictions, compared to one review that did, while 29 reviews did not make any statement on language restrictions.

A limitation to this study is that we only investigated Cochrane systematic reviews, which may stand apart from other reviews because of high standards for the conduct and reporting of literature search. A comparable evaluation of search strategies used in 235 systematic reviews of diagnostic test accuracy published in China before 2011 found that Chinese reviewers had searched Chinese databases (with or without English language databases) in 205 (87%) cases [18].

## Discussion

Our case report highlights that large numbers of publications may be missed when not searching Chinese databases. In a systematic literature survey, we found that Cochrane reviewers searching Chinese databases are an exception, mainly limited to review teams with an author with a Chinese affiliation, and focusing on interventions in complementary or alternative medicine.

A major hurdle to searching Chinese databases is the amount of resources and personnel needed. Systematic review projects often suffer from time and budget constraints, which may conflict with authors’ willingness to maximize the amount of evidence relevant to their clinical question. Searching Chinese databases and extracting data from large amounts of Chinese publications implies having at least one Chinese language reviewer on board or resources to work with biomedical-oriented professional translators. Balk *et al*. found that Google Translate may be helpful in extracting data from non-English literature (Chinese, French, German, Japanese, and Spanish) [19]. Unfortunately, Chinese translations provided the least accurate data in this comparison, with about one out of five items incorrectly extracted more than half of the times when using Google Translate, as compared to extraction performed by a fluent speaker.

The credibility and integrity of the studies that can be additionally included when searching Chinese databases should also be taken into account. We are not aware of any direct way to evaluate the reliability of the data contained in biomedical journals and databases. One can only evaluate indirect evidence, such as evidence of scientific misconduct. A number of cases of scientific fraud and misconduct in China have been reported in the past few years, including plagiarism, falsification, and fabrication of data [20]. This has led to growing concern that those Chinese scandals are only the emerged tip of the iceberg. Chinese scientific authorities and universities seem to have recognized the problem and have increasingly been developing efforts and implementing active policies against research misconduct [21,22]. The issue of research integrity extends far beyond China: a systematic evaluation of all 788 English language research articles retracted from PubMed between 2000 and 2010 found that among all retractions, 33% and 11% originated from the USA and China, respectively [23].

## Conclusions

If we want to include all available evidence in systematic reviews, we need to develop strategies to take into account the huge share of biomedical research published by scientists who write in their native language rather than in English. Otherwise, such valuable information might remain unused by English-speaking evidence-based practitioners and researchers. We advocate that Chinese biomedical research should be more accessible and transparent, to allow better identification of studies published in Chinese. We also suggest to strengthen relationships and collaborative efforts between Chinese-speaking biomedical researchers and non-Chinese-speaking groups, to facilitate the inclusion of Chinese data in systematic reviews when relevant.

## Abbreviations

Anti-CCP: anti-cyclic citrullinated peptide
CBM: Chinese biomedical literature
CMCC: Chinese Medical Current Content
CNKI: China National Knowledge Infrastructure
